# 

**Published:** 1860-05

**Authors:** 


### CONTENTS

OF THE

### Chicago Medical Journal for May, 1860.

### ORIGINAL COMMUNICATIONS.

PAGE.

ARTICLE 1.—A Chapter of Accidents. By David Prince, M. D., Jacksonville, Ill........ 249
ARTICLE 2.—Hysteria. By J. P. Ross, M. D., Attending Physician to City Hospital..... 260
ARTICLE 3.—Wound in the Stomach Treated by Opium. By James Cody, M. D., of Watertown, Wis.... ........................................... 264
Iodine an Antidote to the Venom of the Rattlesnake. By James S. Whitmire, Metamora, Ill. 267
Physiological Incest. By J. J. M. Angrar, M. D., Racine, Wis............ .............. 271

### EDITORIAL.

Book and Pamphlet Notices ............................................272 to 280
Correction—Meeting of Illinois State Medical Society.........  .......................... 279
Removal of the body of Dr. John T. Hunter............................................. 280
Dr. Fisher's Case ........................................................................ 280
Names of the Graduating Class of Rush Medical College................................. 281
De Witt County Medical Society ............................................................ 282
Chlorate of Potash in Mercurial Salivation................................................ 283
Tincture of Iodine Removes Corns.......................................................... 283

### SELECTIONS.

On Union by the First Intention, and Purulent Absorption. By Warren Stone, M. D., Professor of Surgery in the University of Louisiana........................................ 284
Cerebral Symptoms, Independent of Cerebral Diseases. By Charles West, M. D........... 288
Fœtal Auscultation. By Francis Adams, M. D.............................................. 290
Sulphurous Vapor Baths in Chronic Rheumatism. By Dr. G. L. Purdy..................... 295
Case of Abscess of Parotid Gland. By J. Lykins, M. D., Kansas City, Mo................ 297
Funis Presentation. By S. Brandies, M. D., Louisville, Ky................................ 299
The Vaccination Cases at Westford......................................................... 302
Closure of the Fontanelles................................................................. 203
Comparison of the Fatality of some of the Diseases of London, now and in the Seventeenth Century ................................................................................ 304
On the Various Forms of Vulvitis. By Dr. Simpson, Professor of Midwifery in the University of Edinburgh............................................................................. 305
Annual Meeting of the Iowa State Medical Society........................................ 311

